# Additive Manufacturing of Neuromorphic Systems

**DOI:** 10.1002/adma.202504807

**Published:** 2025-07-14

**Authors:** Jiongyi Yan, Yutai Su, James P. K. Armstrong, Andrew Gleadall

**Affiliations:** ^1^ Wolfson School of Mechanical Electrical and Manufacturing Engineering Loughborough University Loughborough LE11 3TU UK; ^2^ School of Mechanics Civil Engineering and Architecture Northwestern Polytechnical University Xi'An 710129 China; ^3^ Department of Translational Health Sciences Bristol Medical School University of Bristol Bristol BS8 1TH UK

**Keywords:** additive manufacturing, artificial neural networks, memristors, neuromorphic computing, transistors

## Abstract

Neuromorphic engineering aims to create brain‐inspired computing systems based on synaptic electronic hardware and neural network software. It combines intelligent materials, advanced processing technology, and computation programs. Additive manufacturing (AM), despite being one of the advanced manufacturing technologies capable of multimaterial processing at the microscale, is not widely applied in neuromorphic hardware fabrication. This gap suggests not only process incompatibility and limited resolution of AM but opportunities to create novel intelligent systems. Here, the state‐of‐the‐art in AM‐printed neuromorphic hardware (synaptic electronics and mechanical systems) is reviewed and discussed the integration of AM techniques with neuromorphic engineering. An outlook of printed neuromorphic systems is provided with low cost and environmental impact but high customizability and design flexibility. With ongoing innovation in AM technologies and materials, AM is envisioned with high throughput and resolution for affordable, scalable, and customizable neuromorphic hardware production. The crossover of AM and neuromorphic engineering facilitates prototyping of brain‐inspired computing architectures for efficient and analog computation. This approach may facilitate various applications including neuromorphic robotics, bionics, and real‐time sensing.

## Graphical abstract

The crossover of additive Manufacturing (AM) and neuromorphic engineering promises a new paradigm in the fabrication of intelligent hardware—one that is sustainable, scalable, cost‐efficient, and customizable. The AM‐printed neuromorphic hardware (electronics and mechanical systems) is examined, and we discussed the technological integration. Upon overcoming process incompatibility and limited resolution issues of AM, 3D‐printed neuromorphic systems may revolutionize intelligent electronics.

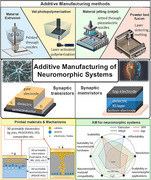

## 1 Introduction

Neuromorphic engineering is an interdisciplinary field that integrates materials science, structural design, and advanced processing techniques to emulate the architecture and functions of biological neural systems. It aims to develop energy‐efficient computing architectures inspired by synapses, the connections between neurons that govern neurotransmission, signal processing, memory‐forming and learning.^[^ ^1^ ^]^ The term ‘neuromorphic’ was proposed by Carver Mead^[^ ^2^ ^]^ to describe neuromorphic analog circuits consisting of transistor networks. Inspired by retinal nerves, he envisioned that neuromorphic systems are inherently scalable and capable of analog computation similar to nervous systems, where information is carried by voltage changes rather than binary states.^[^ ^2^ ^]^ This catalyzed the exploration of neuromorphic computing systems by synaptic transistors and memristors with analog and/or digital resistive switching (RS).^[^ ^3^, ^4^ ^]^

Neuromorphic systems feature architectures comprising interconnected nodes that serve as both processing and memory units, different from the von Neumann architecture^[^ ^5^ ^]^ to mimic how synapses modulate their plasticity (weighted connection strength) based on input signal history and store the information locally.^[^ ^6^ ^]^ Neuromorphic circuits exhibit unique attributes, including scalability, parallelism, adaptability, and low power consumption.^[^ ^7^ ^]^ Neuromorphic electronics include synaptic transistors, memristors, memtransistors, all collectively referred to as artificial synapses.^[^ ^8^, ^9^ ^]^ Transistors are three‐terminal electronics consisting of three electrodes (gate, source, drain), a dielectric layer to insulate the gate, and a semiconductor channel between the source and drain, whereas memristors are two‐terminal electronics with a top electrode, a dielectric layer, and a bottom electrode. Typically activated by bias voltage input, artificial synapses with nonlinear RS can amplify, attenuate, or modulate the signals, where their electrical conductivity and molecular structures can change adaptively.^[^ ^10^ ^]^ The development of artificial synapses has revolutionized neuromorphic engineering, enabling hardware that can not only perform complex computational tasks but also adapt, learn, and evolve. Such adaptability is vital in advanced artificial intelligence (AI) systems (e.g., computer vision) and artificial neural networks (ANNs) designed to self‐learn from their environment and optimize their performance spontaneously.^[^ ^11^ ^]^ Advanced AI chips (e.g., Intel Loihi,^[^ ^12^ ^]^ IBM TrueNorth,^[^ ^13^ ^]^ HP Dot Product Engine)^[^ ^14^ ^]^ exemplify this transformative high‐efficiency computing paradigm.

Currently, the fabrication of neuromorphic hardware still relies heavily on traditional processes, such as photolithography, thin‐film deposition, chemical vapor, and thermal deposition.^[^ ^15^, ^16^ ^]^ These methods, while effective in processing nanoscale electronic units, are exorbitant and rigid, lacking adaptability to novel materials and limited versatility to develop simpler, less labor‐intensive, high‐precision processes.^[^ ^17^, ^18^ ^]^ Replicating intricate 3D structures to emulate the complex connectivity of biological neurons is particularly challenging using these traditional methods.^[^ ^19^ ^]^ Additive manufacturing (AM), which includes 3D printing and other deposition methods, is a class of rapidly evolving manufacturing technologies that can be used to create intricate 3D structures at high resolution with less material and energy waste.^[^ ^20^ ^]^ In particular, AM combines the advantages of high customization, high precision, and environmental friendliness.^[^ ^21^ ^]^ In the context of neuromorphic engineering, AM offers an exciting opportunity to fabricate complex, multimaterial devices to mimic the intricate architectures of neural networks. It may provide a solution to fabricate artificial synapses and even entire neuromorphic circuits with the flexibility to fine‐tune their structural and material properties. AM‐based neuromorphic devices could be applied in wearable intelligent bioelectronics,^[^ ^22^ ^]^ biosensors,^[^ ^23^ ^]^ and robotics.^[^ ^24^ ^]^

However, despite the potential of AM, its application in fabricating neuromorphic hardware remains largely untapped. A few studies have demonstrated inkjet printing for producing neuromorphic devices, while extrusion and laser‐based techniques have not been widely studied. This gap can be attributed to technical challenges of printing intricate architectures at sub‐microscale resolution and the limited number of printable materials that are suited for application. This void leaves numerous opportunities to address limitations faced by traditional manufacturing using AM to produce more affordable, scalable, and customizable devices, paving the way for widespread application of advanced and sustainable neuromorphic systems.

This review focuses on the integration of AM in neuromorphic engineering in terms of materials, technologies, and research opportunities. We first introduce the basis of neuromorphic engineering, including artificial synapses and neuromorphic computing. We then review the state‐of‐the‐art of AM technologies which are suitable for fabricating neuromorphic systems, before reviewing the literature of AM‐based neuromorphic hardware and analyzing the device performance. The novel concept of mechanical neuromorphic systems will be introduced. Finally, we discuss the applications and challenges of AM‐based neuromorphic systems. In this way, we highlight the challenges and opportunities for AM‐based neuromorphic devices and outline how the gap between these two cutting‐edge fields can be bridged to revolutionize intelligent computational paradigms.

## 2 Neuromorphic Engineering

### 2.1 Neuromorphic Electronic Systems

Creating artificial synapses is the first step of physical realization in neuromorphic engineering. Inspired by the biological neurons, artificial synapses emulate neuronal activities at synapses (end structures between neurons):^[^ ^25^ ^]^ release and reception of potential‐induced neurotransmitters by the presynaptic and postsynaptic terminals respectively,^[^ ^26^ ^]^ where the synaptic plasticity can be strengthened, maintained, or weakened.^[^ ^27^ ^]^ Thus, input potentials are computed and postsynaptic output potentials are memorized locally,^[^ ^28^ ^]^ and the spiking dictates synaptic weight modulation.^[^ ^25^ ^]^ This section introduces electronic artificial synapses based on synaptic transistors and memristors.

#### 2.1.1 Synaptic Transistors

##### Mechanisms and Materials

Transistors are three‐terminal electronics that modulate, amplify, and switch input voltage signals, widely used in chips as logic computing units. Synaptic transistors are advanced transistors with two critical features: analog conductance states and Hebbian learning. In response to external stimuli, synaptic transistors exhibit multiple conductance states instead of digital binary states, which emulates the process of synaptic plasticity or weight modulation. For Hebbian learning, one important rule is spike‐timing‐dependent plasticity (STDP) which states the synaptic plasticity is affected by the timing between presynaptic and postsynaptic spikes: it increases if presynaptic spikes fire prior to postsynaptic spikes, whereas the synaptic efficacy decreases if presynaptic spikes fire after the postsynaptic spikes.^[^ ^29^ ^]^ Additionally, frequent spikes cause long‐term potentiation and infrequent spikes cause depression, which are the key to learning and long‐term memory (>hours).^[^ ^30^ ^]^ Synaptic transistors may also exhibit short‐term synaptic plasticity (<seconds): short‐term potentiation and short‐term depression.

**Figure 1:**
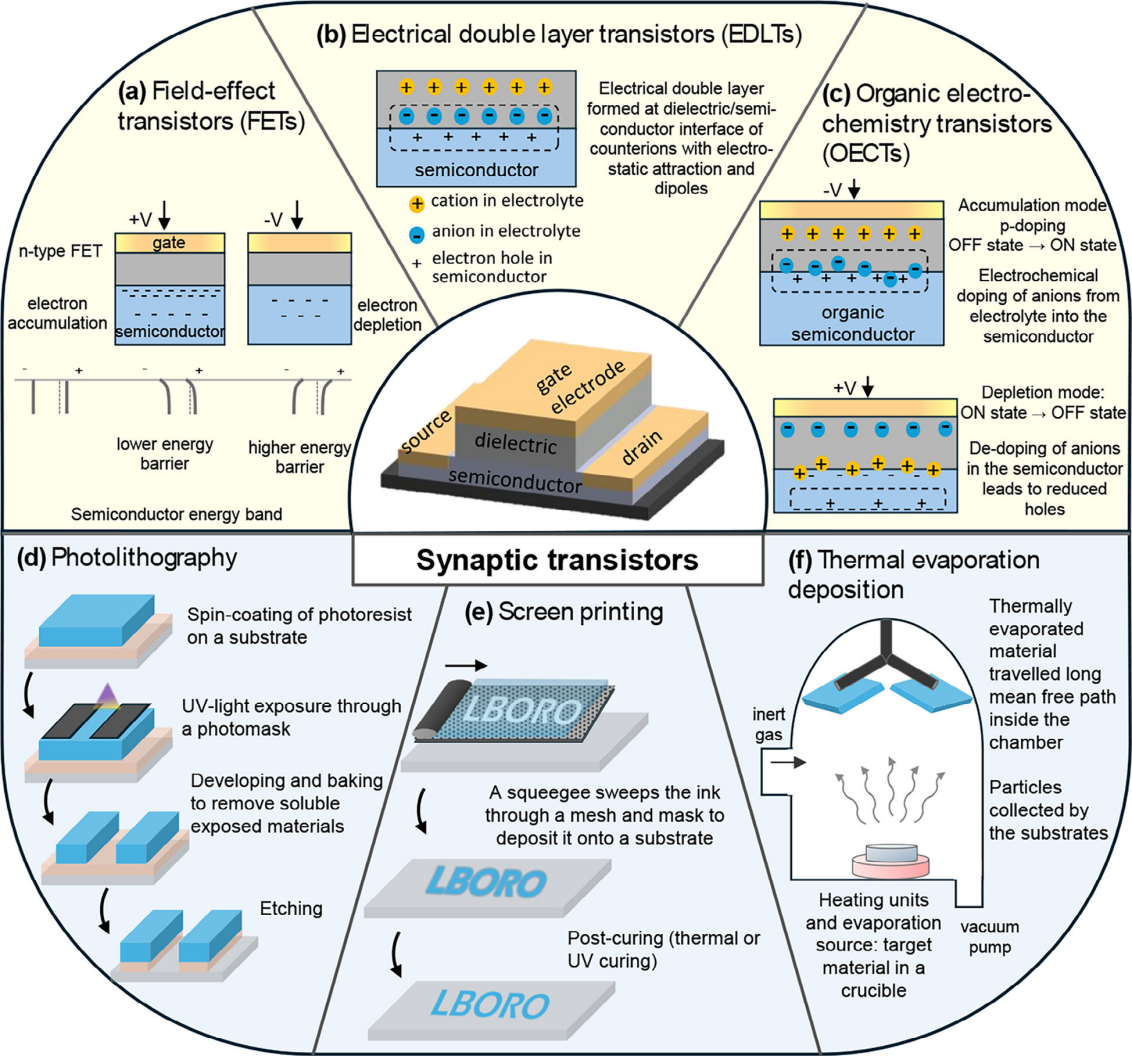
Synaptic transistors used as artificial synapses. Different types of synaptic transistors including a) FETs, b) EDLTs, and c) OECTs. The conventional manufacturing technology includes d) photolithography, e) screen printing, and f) thermal evaporation deposition.

Generally, synaptic transistors can be categorized into field‐effect transistors (FETs), organic electrochemical transistors (OECTs), and electrical double layer transistors (EDLTs) (**Figure** **1**a–c). For FETs, application of a bias voltage on the gate electrode leads to the accumulation or depletion of charge carriers in the semiconductor (electrons for n‐channel, holes for p‐channel).^[^ ^16^ ^]^ However, in OECTs and EDLTs, the dielectric layer is organic and ionically conductive^[^ ^31^ ^]^ where ion migration occurs by applying voltage:^[^ ^32^ ^]^ cations migrate toward the cathode with negative potentials, and the anions toward the anode with positive potentials.^[^ ^33^ ^]^ In OECTs, the mobile ions from the dielectrics are doped into the semiconductor via electrochemistry and result in varying conductance,^[^ ^34^ ^]^ whereas for EDLTs, ions do not penetrate the semiconductor but form an electrical double layer at the interface due to electrostatics.^[^ ^35^ ^]^

As for materials, high‐k metal oxide dielectrics such as hafnium oxide (HfO₂),^[^ ^36^ ^]^ aluminum oxide (Al₂O₃), and zirconium oxide (ZrO₂)^[^ ^37^ ^]^ are used in FETs for higher capacitance. Organic electrolytes, including poly(ethylene oxide), ionogels (e.g., ethyl‐3‐methylimidazolium bis(trifluoromethylsulfonyl)imide with poly(ethylene glycol) diacrylate)^[^ ^38^ ^]^ and hydrogels (e.g., cellulose, gelatin, polyacrylamide)^[^ ^10^ ^]^ were studied. Oxide‐based semiconductors, such as indium‐gallium‐zinc oxide (IGZO),^[^ ^39^ ^]^ zinc oxide (ZnO), and perovskite oxides^[^ ^40^ ^]^ are common due to excellent electrical properties and processing compatibility. 2D materials, including graphene,^[^ ^41^ ^]^ hexagonal boron nitride (h‐BN),^[^ ^42^ ^]^ and molybdenum disulfide (MoS₂)^[^ ^43^ ^]^ are of interest due to the high mobility of their charge carriers. Organic semiconductors include poly(3,4‐ethylenedioxythiophene): poly(styrene sulfonate) (PEDOT: PSS),^[^ ^44^ ^]^ P3HT,^[^ ^45^ ^]^ PDVT,^[^ ^46^ ^]^ pentacene and tetracene,^[^ ^47^, ^48^ ^]^ and these materials with π‐conjugation show good conductivity, which is similar for carbon‐based materials (carbon nanotubes,^[^ ^49^ ^]^ fibers).^[^ ^50^ ^]^ PEDOT: PSS is versatile and compatible with various AM processes. Due to mixed ionic‐electronic conduction and doping with high hole density,^[^ ^51^ ^]^ it can serve as an electrode material due to intrinsic doping and charge carrier delocalization.^[^ ^49^ ^]^ In OECTs, positive voltage drives electrolyte cations toward the channel and compensates the PSS polyanions, causing PEDOT de‐doping and lowered conductivity,^[^ ^52^ ^]^ and direct doping of mobile ions without an electrolyte has been explored.^[^ ^53^ ^]^ In addition, hydrogels^[^ ^10^ ^]^ and ionogels^[^ ^45^, ^54^ ^]^ with intrinsically good ionic conductivity, biodegradability, and flexibility^[^ ^55^, ^56^, ^57^ ^]^ are good dielectric materials for AM‐based synaptic devices.

##### Manufacturing and Processing

The conventional processing techniques of complementary metal‐oxide‐semiconductor transistors are well‐developed.^[^ ^6^ ^]^ AM, as a novel alternative method, will be discussed in detail in Section 3.1. Spin‐coating and photolithography are standard nanofabrication for integrated circuits.^[^ ^16^ ^]^ This process transfers designed shapes from a photomask onto a substrate using light (Figure 1d). Typically, a photoresist layer with sub‐micron thickness is spin‐coated onto a substrate on top of a silicon wafer.^[^ ^58^ ^]^ UV light is shone through a photomask, causing photochemical reactions and structural changes of the photoresist that can be further baked. For positive photoresists, the light‐exposed areas become more soluble in the developer solution. After development, the exposed soluble photoresist is removed and the surface is etched, leaving only the unexposed areas.^[^ ^59^ ^]^ With precise control of the light wavelength, optical aperture, and focal length, photolithography can achieve <10 nm resolution.^[^ ^60^ ^]^ Further miniaturization can be achieved through the use of phase‐shifting photomasks,^[^ ^61^ ^]^ extreme UV,^[^ ^62^ ^]^ electron beam lithography,^[^ ^63^ ^]^ and secondary sputtering lithography.^[^ ^15^ ^]^ Inkjet printing and screen printing are common techniques, especially for organic transistors, due to their relatively easy processability and compatibility with flexible thin‐film transistors.^[^ ^64^ ^]^ Screen printing is a contact‐based printing technique whereby inks are printed through a mesh or stencil onto a substrate^[^ ^65^ ^]^ (Figure 1e). It is suitable for large‐scale production of electronics due to high throughput and low cost, however, it offers low resolution and generates more waste due to the lower precision of material deposition. Thermal evaporation is a physical vapor deposition where a target material evaporates at high temperature and forms a thin layer of coating on a substrate (Figure 1f). It is suitable to process polymers, ceramics, and metals at sub‐micron resolution. However, precise control of structure configuring is problematic in thermal evaporation.

#### 2.1.2 Memristors

**Figure 2:**
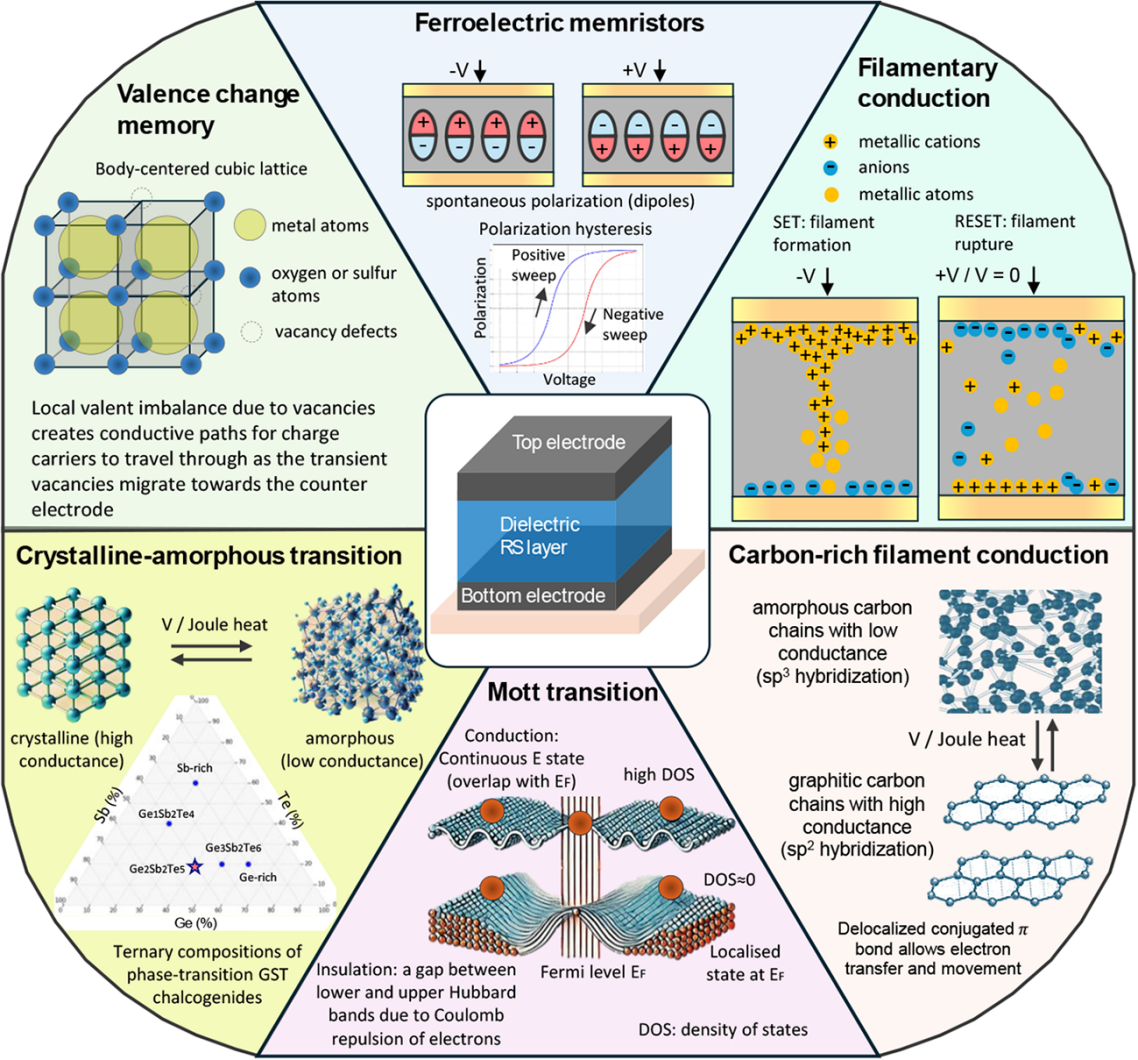
Different types of memristor mechanisms, including valence change memory, ferroelectric memory, filamentary conduction, crystalline‐amorphous phase transition, Mott transition, and carbon‐rich filament conduction.

Memristors are memory resistors, a fundamental circuit element based on the non‐linear relationship between charge and flux theorized by Chua.^[^ ^3^, ^66^ ^]^ Memristors exhibit varying resistance dependent on the charge flow history, which is identified by voltammetric hysteresis. One of the earliest memristors was invented by HP Labs:^[^ ^67^ ^]^ 5‐nm‐thick titanium oxide films exhibited voltage–current hysteresis that conferred memristance due to doping and drifting of oxygen vacancies. Different memristive behaviors and mechanisms have since emerged.^[^ ^17^, ^68^, ^69^ ^]^ Memristors are natural candidates for artificial synapses because the memristance modulation affected by input voltage resembles biological synaptic plasticity.^[^ ^69^, ^70^, ^71^ ^]^ Memristors can be categorized by memristive mechanisms, which include vacancy valence change, crystalline‐amorphous phase‐transition, metal‐insulator Mott transition, ferroelectric polarization, filamentary conduction, and capacitive coupling^[^ ^72^ ^]^ (**Figure** **2**).

Valence change memristors include binary metal oxides and perovskites, where oxygen or sulfur vacancies modify the local electronic structure.^[^ ^73^ ^]^ Localized lattice defects change the valence and transient vacancy migration toward the counter electrode with an external voltage, during which vacancies form pathways for charge carriers to flow.^[^ ^17^ ^]^ Crystalline‐amorphous phase‐transition memristors include chalcogenide compounds, such as germanium—antimony–tellurium,^[^ ^74^ ^]^ which undergo a reversible Joule‐heat‐induced transition from a high‐resistance amorphous state to a low‐resistance crystalline state when above a critical recrystallization temperature.^[^ ^75^ ^]^ Mott transition occurs in Mott insulators (e.g., cobalt oxide), which typically exhibit insulation at low temperatures due to strong electron–electron interactions described by the Hubbard model.^[^ ^76^ ^]^ In this process, applied electrical fields induce damping and cause the localization of electrons, and therefore the material displays a metal‐to‐insulator transition.^[^ ^76^ ^]^ Ferroelectric memristors are driven by ferroelectric polarization switching, where an applied electrical field switches the direction of spontaneous polarization (binary magnetized spin states) and further alters Schottky barriers and tunnelling probability.^[^ ^77^ ^]^ The magnetic tunnelling effect occurs in saturated parallel magnetization (magnetic tunnel junctions), resulting in high tunnelling conductivity.^[^ ^78^ ^]^ Filamentary conduction, namely electrochemical metallization, occurs when employing electrochemical redox reactions of the top electrode (active metals such as Ag and Cu).^[^ ^79^ ^]^ With applied voltage, metal atoms are oxidized into cations, which accumulate to form conductive nano‐filaments that later migrate to the counter electrode, facilitating conductive pathways for charge carriers.^[^ ^80^ ^]^ Non‐metallic filaments also appear in valence change memristors (e.g., TiO₂,^[^ ^81^ ^]^ HfO₂),^[^ ^82^ ^]^ where vacancy drift locally reduces oxide and form vacancy filaments or sub‐oxide filaments,^[^ ^83^ ^]^ and metallic conductive filaments may also exist from overdoping.^[^ ^84^ ^]^ Another filament mechanism was reported as carbon‐rich filament conduction, where an applied bias induces local Joule heat and chain state transition from amorphous with high resistance (sp^3^ hybridization)^[^ ^80^ ^]^ to graphitic with low resistance (sp^2^ hybridization).^[^ ^85^ ^]^ The graphitization encourages carbon‐rich filaments that act as conductive pathways for charge carriers. The capacitive coupling theory is used to explain memristive behavior, whereby dielectric properties and capacitance may change and affect the distribution and migration of charge carriers^[^ ^72^ ^]^ therefore leading to resistance changes.^[^ ^86^ ^]^

#### 2.1.3 Synaptic Transistors versus Memristors

Compared to three‐terminal transistors, two‐terminal memristors have a simpler electrode‐dielectric‐electrode structural configuration, enabling straightforward layerwise manufacturing by sputtering, AM, spin‐coating, and deposition.^[^ ^6^, ^87^ ^]^ This simplicity leads to lower manufacturing cost and high scalability for memristors in high‐density compact‐crosspoint crossbars, where they can be also vertically integrated above CMOS logic circuits.^[^ ^17^, ^88^ ^]^ Transistors require multimaterial stacking and complex masking, deposition, and alignment steps, although highly optimized in well‐established CMOS processes for scaled large‐area fabrication. Regarding implementation, memristors are passive elements without a gating terminal, making their synaptic weight modulation dependent solely on voltage‐history between electrodes,^[^ ^73^ ^]^ whereas the gate electrode in transistors provides separate selectivity and direct control, thus reducing sneak‐path risks.^[^ ^89^ ^]^ Multi‐gated transistors have been studied to improve the weight update linearity and accuracy.^[^ ^90^, ^91^ ^]^ Memristor readout is essentially Ohm's Law, but risking inadvertent altering and retention of resistance state if the read voltage exceeds a threshold, especially for non‐volatile memories.^[^ ^73^, ^92^ ^]^ Contrarily, transistor readout is non‐destructive, where the weight is encoded as a threshold voltage shift (via trapped charge or remnant polarization), so the read does not erase the stored charge or polarization.^[^ ^93^ ^]^ As for system scalability, while nanoscale memristors enable high‐density analog computing and 3D architectures, nonlinear and asymmetric weight updates complicate algorithmic implementations.^[^ ^71^ ^]^ Conversely, large‐area transistors provide reliable read–write operations but cell sizes trade off density.^[^ ^9^ ^]^

### 2.2 Neuromorphic Mechanical Systems

Mechanical neuromorphic systems are based on mechanical structural components, usually AM‐based, which could emulate neuronal functions of learning and computing under mechanical stimuli.^[^ ^94^ ^]^ Similar to electronic memristors, mechanical memristors exhibit memory effects to retain historical information of force input. This behavior enables both threshold switching, akin to digital binary states, and analog memory effects, analogous to continuous changes in conductance. This key functionality lies in the dynamic modulation of mechanical properties, such as stiffness.^[^ ^94^ ^]^ When subjected to varying mechanical loading, mechanical memristors can exhibit hysteretic changes in their compliance or strain response, which depend on the input history ($\frac{dF}{d\delta }$).^[^ ^94^ ^]^ This value is notationally the same as stiffness but represents the memristive property that captures the relationship between the force *dF* and displacement *d*δ.^[^ ^94^ ^]^ Structures, such as architected metamaterials or lattice‐based systems, employ mechanisms that adjust their stiffness dynamically. For example, nonlinear deformation (buckling or viscoelastic creep) may introduce hysteretic stiffness changes. Geometrical nonlinearity (auxetic or bistable metamaterials)^[^ ^95^ ^]^ may also enable such programmable stiffness.

### 2.3 Neuromorphic Computing

Neuromorphic computing relies on neuromorphic hardware integrated in a crossbar array circuit to implement vector matrix multiplication, a foundational operation in neural networks.^[^ ^5^ ^]^ For a single artificial synaptic device, the STDP and weight modulation represent how input signals can be processed and learned on one node of a neural network. In a crossbar array, artificial synapses are arranged in a matrix and usually with a gate electrode beam array on the top and a bottom electrode beam on the bottom,^[^ ^9^ ^]^ whose conductance represents the synaptic weight. Input voltage is applied to rows (*V_i_*), and output currents (*I*) are measured at the columns, according to Ohm's Law.^[^ ^96^ ^]^ The summation of these currents along each column, dictated by Kirchhoff's Law, produces the dot product of the input voltage vector and the conductance matrix *G* to show synaptic weight, thereby enabling analog vector‐matrix multiplication.^[^ ^79^ ^]^ Initially, the conductance values are set randomly or based on former information^[^ ^97^ ^]^. Training is achieved by applying iterative voltage inputs, measuring output currents, and adjusting synaptic weights based on errors (the difference between the actual output and desired output) or timing relationships.^[^ ^98^ ^]^

Learning rules, such as STDP, enable hardware‐level updates by modifying conductance based on the timing of pre‐ and post‐synaptic signals. For learning based on gradient descent (software optimization to minimize errors by gradient of the error function with respect to the synaptic weights), additional digital processing units or hybrid designs might be required.^[^ ^94^ ^]^ The STDP also facilitates spiking neural networks (SNNs) for individual nodes to process and learn temporal and spatial input patterns.^[^ ^99^ ^]^ The local learning dynamics of SNNs naturally enable gradient‐free optimization directly at the hardware level, reducing reliance on external processors and further enhancing energy efficiency.^[^ ^100^ ^]^ Effective signal encoding in hardware‐implemented SNNs is crucial to accurately represent data as spike trains. There are three common SNN encoding strategies. The rate‐coding involves encoding analog inputs into spike frequencies, with greater inputs producing higher spike rates^[^ ^101^ ^]^ exemplified by BrainScaleS.^[^ ^102^ ^]^ This typically employs Poisson spike trains generated by comparing analog inputs with random thresholds at each timestep.^[^ ^101^ ^]^ Temporal coding encodes the precise timing of individual spikes (e.g., logarithmic temporal coding encodes an activation value with a binary string with a predefined length, and the active spike number grows logarithmically).^[^ ^103^ ^]^ Finally, event‐based sensors use direct and inherent temporal encoding (e.g., dynamic vision sensors output spike events asynchronously, triggered by event‐driven changes in image pixel intensity).^[^ ^101^ ^]^

To mitigate a major challenge of sneak path currents (unintended current flows through unselected devices that impair computational accuracy) in crossbar arrays, selector devices with high nonlinearity or asymmetric *I−V* characteristics are often integrated at junctions to enhance the scalability and reliability.^[^ ^98^ ^]^ Innovative magnetoresistive crossbars with resistance summation have been designed to reduce power consumption and increase precision.^[^ ^96^ ^]^ Neuromorphic computing systems allow computations to occur simultaneously in parallel and large scale.^[^ ^92^ ^]^ They emulate the functionality of biological neural networks, providing a foundation for scalable, energy‐efficient, and adaptive AI systems.^[^ ^104^ ^]^

## 3 AM in Neuromorphic Engineering

### 3.1 AM Techniques and Materials

AM includes material extrusion, vat photopolymerization (VPP), powder bed fusion (PBF), material jetting, binder jetting, sheet lamination, and direct energy deposition, according to ISO/ASTM 52900.^[^ ^105^ ^]^ Materials and mechanisms differ for techniques.^[^ ^106^ ^]^ Here we discuss AM techniques suitable for manufacturing neuromorphic devices: material extrusion, VPP, inkjet printing, and PBF. Other AM techniques (direct energy deposition, sheet lamination, and binder jetting) are yet to be developed for manufacturing functional electronics^[^ ^64^ ^]^ mostly due to limitations in materials and resolutions.

#### 3.1.1 Material Extrusion

**Figure 3:**
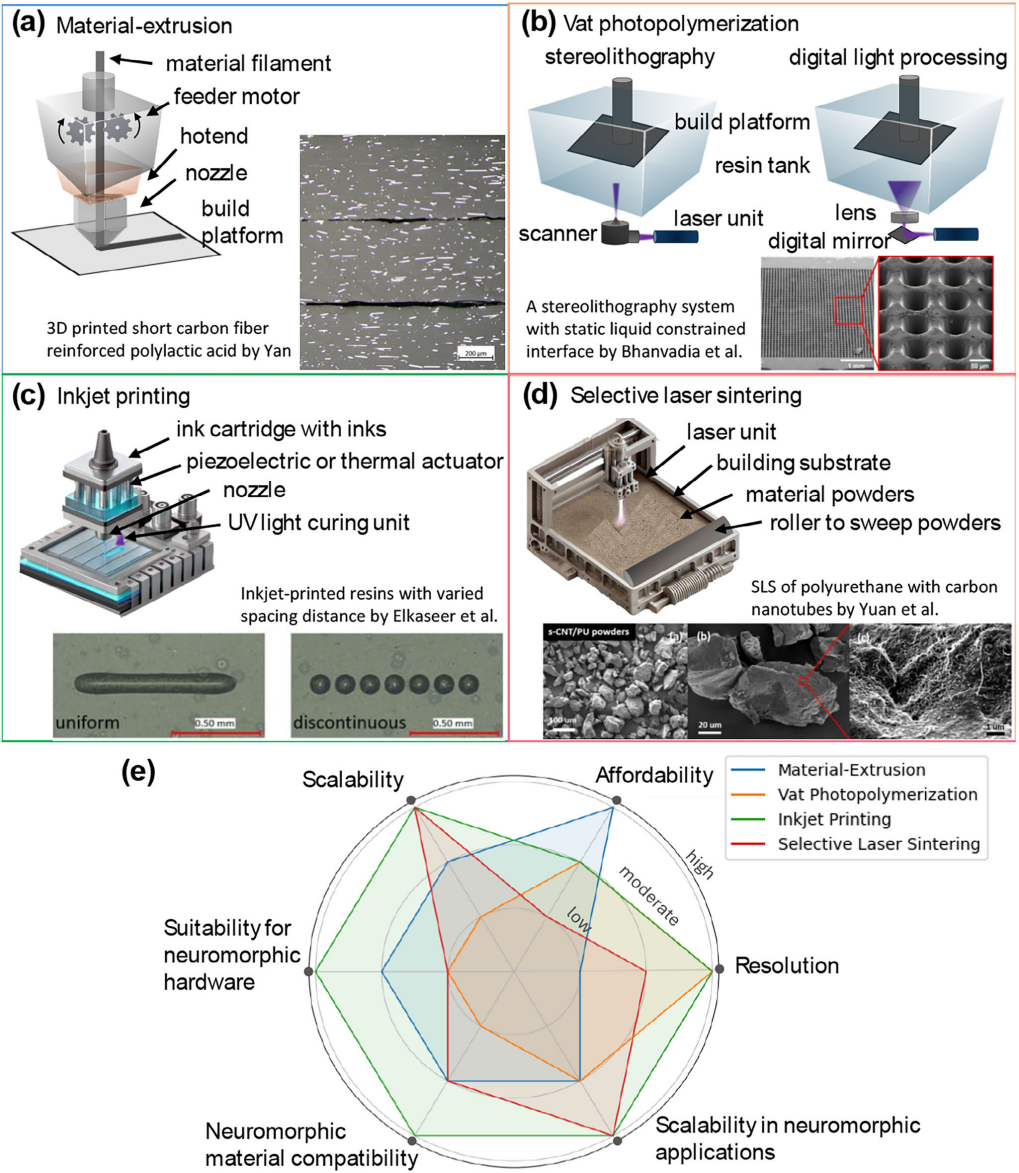
Four main additive manufacturing techniques: a) extrusion‐based printing, b) vat photopolymerization, c) inkjet printing, and d) selective laser sintering. e) A comparison between these four techniques in terms of their performance and suitability for neuromorphic hardware production. Micrographs in a) are original work, b) are reproduced under the terms of the Creative Commons CC BY license.^[^ ^131^ ^]^ Copyright 2021 Springer Nature, c) are reproduced under the terms of the Creative Commons CC BY license.^[^ ^145^ ^]^ Copyright 2022 MDPI, d) are reproduced with permission.^[^ ^149^ ^]^ Copyright 2018 Elsevier.

Material extrusion AM is a process where material is extruded from a nozzle into individual extruded lines and built layer by layer. It is often referred to as fused deposition modelling (FDM) when used for thermoplastics, direct ink writing when used for gels or pastes, and other names when used for biomaterials, concrete, and various other extrudable materials. In FDM, thermoplastic polymers are melted and extruded through a nozzle (**Figure** **3**a) and then solidify to form a stable structure onto which subsequent polymer can be deposited. For other materials, they typically need carefully controlled rheology that means smooth extrusion but also structural integrity to support their own weight and subsequent layers’ weight. Extrusion AM benefits from low cost, high production speed, and easy operation.^[^ ^107^ ^]^ It supports the widest range of materials of all processes, although metals and ceramics typically need significant postprocessing operations. However, the resolution is lower than other processes and, therefore a limiting factor for printing artificial synapses. The resolution of extrusion‐based printing is limited by the nozzle size (typically ≥150 µm).^[^ ^107^ ^]^ The *Z*‐axis stepper motors can be of high resolution (10 µm),^[^ ^108^ ^]^ and low layer heights are possible (<50 µm).^[^ ^109^ ^]^ Hunde et al.^[^ ^110^ ^]^ extrusion‐printed multilayer PEDOT: PSS films with <150 nm thickness, used as perovskite solar cells, which suggests the potential of submicron extrusion printing to be further explored. To increase material functionality, conductive materials including composites,^[^ ^111^, ^112^ ^]^ organic conductive materials (e.g., PEDOT: PSS)^[^ ^51^, ^113^ ^]^ and reinforcements^[^ ^114^ ^]^ (e.g., graphene oxide, carbon nanotubes, and metal powders)^[^ ^115^ ^]^ have been studied. Functional particles may change the rheological properties, which can impact the material printability(e.g., short carbon fibers are most printable at layer heights ≥200 µm).^[^ ^116^, ^117^ ^]^ Reducing nozzle size may impact material flow, and compounding materials to increase functionality may complicate rheology and printability.^[^ ^118^ ^]^

Another concern of extrusion‐based AM is surface roughness. Microscale roughness of the printed surface is commonly present (typically <5 µm for single extrudates)^[^ ^119^, ^120^ ^]^ which can diminish the electrical properties.^[^ ^121^, ^122^ ^]^ Despite such high smoothness compared to powder‐based processes, it is still insufficient for transistors and memristors, as dielectric properties and charge carrier mobility are sensitive to surface roughness. While nano‐porosity can enhance ion migration,^[^ ^123^ ^]^ in general, smoother surfaces of electrodes and dielectrics are favored as they allow charge carriers or ions to travel without scattering or leakage due to structural imperfections.^[^ ^124^, ^125^ ^]^ Annealing,^[^ ^36^ ^]^ spray deposition and chemical treatments^[^ ^126^ ^]^ can improve the surface smoothness and RS performance.

#### 3.1.2 Vat Photopolymerization

Vat photopolymerization (VPP) uses photopolymer resins that are selectively cured by light‐activated polymerization to form 3D structures (Figure 3b). Different hardware can be used for the VPP process: a laser, a projector, and a screen. Stereolithography refers to using a laser source to initiate local polymerization within a focal area, whereas digital light processing uses projected digital light to cure the resin across a whole plane.^[^ ^127^ ^]^ The print quality and properties depend on the resin, initiators, additional photoabsorbers, and exposure conditions. VPP features relatively smooth surface finishing, good resolution (layer height < 50 µm and spot size < 150 µm)^[^ ^128^ ^]^ and controlled post‐curing for single‐material manufacturing. Despite, it has not been adopted for processing neuromorphic hardware due to material incompatibility and inability to process multimaterial architectures. Since most photopolymers are insulators,^[^ ^111^ ^]^ increasing the conductivity with metal particles and conductive polymers has been studied.^[^ ^129^, ^130^ ^]^ Recent innovations in continuous and micro‐VPP bring new opportunities. High resolutions were achieved in stereolithography modified with inert immiscible liquids (<20 µm)^[^ ^131^ ^]^ and HD‐DVD optical pickup units (<250 nm).^[^ ^132^ ^]^ Two‐photon lithography, in which two photons are absorbed simultaneously at the central intensity of a focused near‐infrared laser, can also be used to cure photopolymers.^[^ ^127^ ^]^ Combined with high numerical aperture lenses, this method creates localized nano‐voxel photopolymerization at resolution <60 nm.^[^ ^128^ ^]^ Continuous liquid interface production employs an oxygen‐permeable zone below the resin to avoid undesired oxygen inhibition and to accelerate the process at resolution <30 µm.^[^ ^133^, ^134^ ^]^ Building on these advances, we may propose a conceptual blueprint integrating continuous liquid interface production with a moving film substrate across multiple digital light processing units, similar to on‐film roll‐to‐roll production.^[^ ^135^ ^]^ Each unit could deposit and cure distinct functional resins to create multimaterial, multilayer neuromorphic architectures. However, this approach may raise its own challenges: layer adhesion, prevention of cross‐contamination, and curing variations.

#### 3.1.3 Inkjet Printing

Inkjet printing is a material jetting method, where semi‐solid materials such as photopolymers are selectively ejected through a nozzle onto a substrate^[^ ^136^ ^]^ (Figure 3c). Material rheology is a vital factor to dictate flow through the nozzle under piezoelectric or thermal actuation and subsequent solidification for structural integrity.^[^ ^65^ ^]^ Two main advantages (high feature resolution and flexible multimaterial selection within a single part) make inkjet printing promising for neuromorphic device fabrication. The layer thicknesses of less than 20 nm have been achieved^[^ ^37^ ^]^ which is ideal for high energy efficiency of artificial synapses, as the mobility of charge carriers and ions reduces drastically with increasing thickness.^[^ ^39^ ^]^ Inkjet printing can accommodate a wide range of materials, including conductive inks, semiconductors, polymers, dielectric, and functional composite materials.^[^ ^137^, ^138^ ^]^ This enables complex multimaterial architectures to be produced at relatively low cost and high precision. For instance, conductive inks based on silver nanoparticles or carbon‐based nanomaterials have been inkjet‐printed as electrodes^[^ ^139^, ^140^ ^]^ while organic semiconductors and polymeric electrolytes have also been used.^[^ ^139^, ^141^ ^]^ Recent studies have reported the inkjet printing of 2D materials (e.g., graphene, chalcogenides, MXene).^[^ ^142^ ^]^ For example, Molina‐Lopez et al.^[^ ^48^ ^]^ printed a synaptic organic FET with ionic PEDOT: PSS, carbon nanotubes, and fluorinated polymer dielectrics. However, several concerns should be considered: the material viscosity, surface tension, and particle size distribution must be optimized to ensure proper jetting and patterning.^[^ ^65^, ^142^ ^]^ Uncontrolled formulation and flow may result in nozzle blockage^[^ ^143^ ^]^, coffee ring effects, layer crosstalk, and misalignment.^[^ ^87^, ^144^ ^]^ In addition, the printing parameters, droplet sizes, substrate wettability, and drying process should be well‐controlled.^[^ ^145^ ^]^

#### 3.1.4 Powder Bed Fusion

PBF uses a laser to locally sinter powders (e.g., metallic, ceramic, polymeric) on a powder bed before a sweeper blade or roller lays down a new layer of powder and the process continues layer by layer to form 3D structures (Figure 3d). PBF struggles to achieve sub‐micron resolution, as it typically uses powder particles >20 µm^[^ ^146^ ^]^ and infrared lasers with spot sizes of >50 µm.^[^ ^147^ ^]^ The process is commonly valued for its ability to print intricate geometries because the bed of unsintered powder acts as support for intricate structures. However, when the laser heats local particles for coalescence, nearby particles can partial melt or deform, which not only reduces the printing resolution but also increases the surface roughness, typical root‐mean‐square ≈20 µm.^[^ ^148^ ^]^ Fusion uniformity and thermal effects remain the main challenges. As for the material selection, PBF is not highly suitable to process semiconductors and conductive polymers due to thermal degradation. Nevertheless, by precise control of the laser and careful formulation of materials, it has been used to print conductive carbon‐polymer composites,^[^ ^149^, ^150^ ^]^ chalcogenide semiconductors,^[^ ^151^ ^]^ and dielectric composites.^[^ ^152^ ^]^ Roy et al.^[^ ^153^ ^]^ reported micro‐laser‐sintering of nanoparticles at <5 µm resolution. PBF, albeit with resolution and material issues, may be useful as a complementary technique. Ko et al.^[^ ^154^ ^]^ reported PBF of inkjet‐printed gold nanoparticles in FETs and PBF‐ablation of all‐inkjet‐printed FET.^[^ ^140^, ^155^ ^]^ Theoretically, PBF of nanoparticles to form thin electrode layers or RS layers requires lower energy, with corresponding improvements in the print resolution. For example, direct laser writing of nanoparticles has been applied to produce memristive electronics.^[^ ^156^ ^]^ However, PBF is not suitable for multimaterial processing, as the parts are submerged in the dispersed powders and changing powders is not feasible. Thus, PBF has not been singularly used for neuromorphic hardware.

#### 3.1.5 Comparison between AM Techniques

We compared the four main AM techniques in terms of their capability and process translatability for neuromorphic hardware production (**Table** **1**). Overall, inkjet printing is the most advantageous technique due to its ability to print multimaterial multilayers at high resolution with moderate cost, despite laborious material and process control (Figure 3e). While VPP and PBF generally do not support multimaterial processing, and scalability is limited by batch flows thus not yet ready for functional electronics. Even if the resolution issue is overcome, questions remain about the system compatibility and material functionality for complex multimaterial architectures. Extrusion‐based AM, although suitable for multimaterial multilayer structures, remains a less‐common choice for low‐cost, structural components, such as sensors and capacitive devices. It has the potential to scale up for neuromorphic hardware or combine with other techniques once the resolution issue is overcome. AM is a rapidly changing research discipline, and innovations toward higher resolution and functionality may open new opportunities in the future.

**Table 1:** Comparison of major AM techniques (extrusion, vat‐photopolymerization, inkjet printing, and selective laser sintering) and analysis of their compatibility for neuromorphic hardware.

	Extrusion	Vat photopolymerization	Inkjet printing	Powder bed fusion
Materials	Polymers, ceramics, metals, composites	Photopolymers, composites	Polymers, ceramics, metals, composites	Polymers, ceramics, metals, composites
Resolution	Low 50–300 µm^[^ ^109^ ^]^	High 10–100 µm^[^ ^131^ ^]^	High 2—50 µm^[^ ^157^ ^]^	Moderate 50–200 µm^[^ ^158^ ^]^
Cost	Printers: low Materials: low	Printers: moderate Resin: low	Printers: moderate Inks: high	Printers: high Material: high
Precision	Moderate (±50 µm)^[^ ^126^ ^]^	High (±10 µm)^[^ ^131^ ^]^	Very high (±1 µm)^[^ ^145^ ^]^	Moderate (±50 µm)^[^ ^148^ ^]^
Suitable substrates	Rigid and flexible (e.g., glass, polymers)	Mostly rigid (e.g., metals)	Rigid and flexible (e.g., polymers, glass)	Rigid (e.g., same as powder material)
Scalability	Moderate (good for prototyping and low throughput)^[^ ^159^ ^]^	Low‐to‐moderate (fast build rate but a batch process)^[^ ^160^ ^]^	High (suitable for high‐throughput small‐sized electronics)^[^ ^161^ ^]^	Low‐to‐moderate (high‐density part nesting but a batch process)^[^ ^147^ ^]^
Post‐processing	Support removal, surface finishing	Support removal, washing, post‐curing	Sintering for metals and ceramics, drying, curing	Powder removal, support removal, heat treatments, finishing
Suitability to produce neuromorphic hardware	Moderate: low resolution but good for multimaterial structures	Low: high resolution but limited multimaterial capabilities	High: high resolution, excellent multimaterial capabilities	Low: limited resolution and unable to produce multimaterial parts
Material compatibility for neuromorphic hardware	Excellent materials range but limited commercial availability	Limited (photopolymers require modification for functionality)	Excellent materials range and commercial availability	Moderate (semiconductors and dielectrics)
Scalability in neuromorphic applications	Scalable with hardware replication but not widely done commercially	Limited to batch processing but potential scalability for sheet substrates	High: can scale up to sheet‐based electronics in large quantities	Limited to batch processing

### 3.2 AM of Neuromorphic Electronics

#### 3.2.1 AM of Synaptic Transistors and Memristors

Inkjet printing of transistors appeared in the early 2000s and was shown to be suitable for generating solution‐processed organic FETs and OECTs. Sirringhaus et al.^[^ ^162^ ^]^ reported an organic FET with inkjet‐printed PEDOT: PSS electrodes on top of a polyimide film and showed high field‐effect mobility. The authors verified the use of inkjet printing to create high‐resolution transistors (>10 µm droplet radius). Both Ko and Sekitani et al.^[^ ^154^, ^157^ ^]^ reported organic FETs with inkjet‐printed Ag nanoparticles as electrodes (width ≈2 µm, thickness ≈30 nm) using a sub‐femtoliter inkjet printer. Their FETs had low operation voltage and high ON/OFF ratios, suitable for miniaturized electronics. These studies leveraged AM for low‐cost, material‐saving, and high‐resolution organic transistors, and they also underpinned the further development of inkjet‐printed electronics.

**Figure 4:**
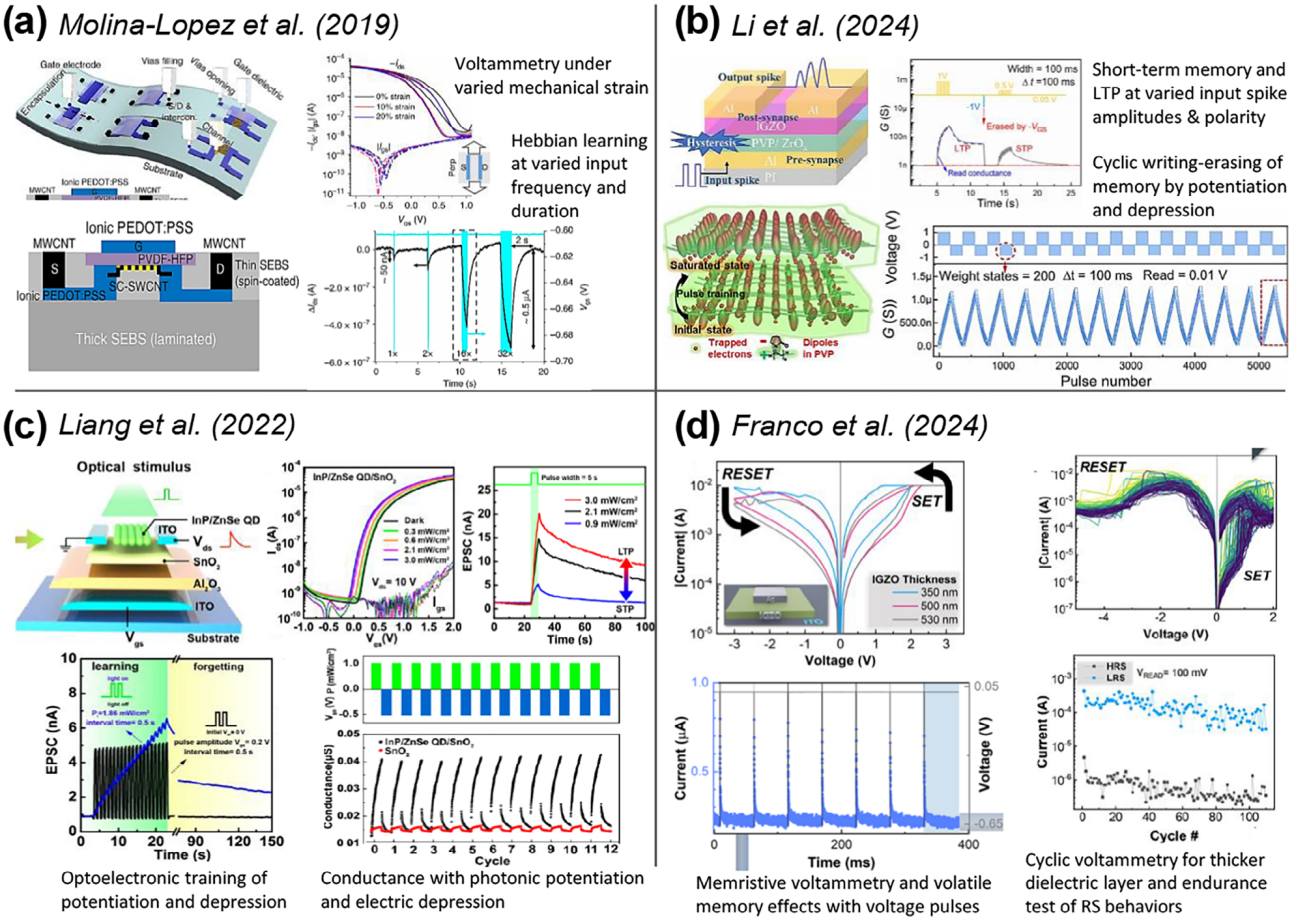
Examples of inkjet‐printed synaptic transistors and memristors. a) A stretchable synaptic EDLT with synaptic behaviors in response to varied voltage input, reproduced with permission.^[^ ^49^ ^]^ Copyright 2019 Springer Nature. b) A synaptic FET with polar‐electret dielectrics with long‐term memory, reproduced with permission.^[^ ^169^ ^]^ Copyright 2024 ACS. c) An optoelectronic heterojunction FET with light‐programmable memory effects, reproduced with permission.^[^ ^170^ ^]^ Copyright 2022 ACS. d) A memristor with volatile and non‐volatile memory, reproduced under the terms of the Creative Commons CC BY license.^[^ ^39^ ^]^ Copyright 2024 Springer Nature.

The early 2010s saw reports of numerous all‐inkjet‐printed transistors. These included a combination of (Ag‐nanoparticle electrodes, poly(4‐vinylphenol) dielectrics, and flexink semiconductors,^[^ ^141^, ^163^, ^164^, ^165^, ^166^ ^]^ as well as pentacene semiconductors.^[^ ^139^, ^167^, ^168^ ^]^ Research into novel inkjet‐printed transistors has been limited by materials, resolution, and stability. Successful jetting of droplets depends on cartridge systems, nozzle sizes, and material rheology. Though sub‐femtoliter printers have emerged, the inclusion of nanoparticles into the ink can lead to unstable jetting and nozzle blockage. However, with the recent surge in neuromorphic AI applications, inkjet printing of neuromorphic transistors and memristors regained attention. Molina‐Lopez et al.^[^ ^49^ ^]^ reported a synaptic EDLT consisting of inkjet‐printed PEDOT: PSS electrodes, a carbon nanotube semiconductor, and a fluorinated copolymer dielectric, which showed high electrical mobility and a high ON/OFF ratio (**Figure** **4**a). More importantly, they demonstrated synaptic behaviors, with the electrical current varying in response to the input voltage amplitude, frequency, and duration. This demonstration opened the path toward inkjet‐printed synaptic electronics. Kim et al.^[^ ^37^ ^]^ reported a synaptic EDLT with an inkjet‐printed indium‐tin‐oxide semiconductor (15 nm thickness, 400 µm width, 80 µm length). This device showed long‐term and short‐term plasticity with a high ON/OFF ratio. Carey et al.^[^ ^43^ ^]^ reported FETs with inkjet‐printed 2D‐MoS_2_ semiconductors acting as a complementary logic inverter in integrated circuits. Chen et al.^[^ ^37^ ^]^ reported a FET array containing inkjet‐printed PVDT semiconductors and studied droplet spacing effects. They also showed short‐term and long‐term plasticity to mimic one‐to‐more neuron functions. Li et al.^[^ ^169^ ^]^ reported a FET based on inkjet‐printed polar‐electret dielectrics and achieved high amplified synaptic weight modulation due to microfluid‐induced dipole orientation (Figure 4b). It also showed short‐term and long‐term plasticity, as well as STDP, and achieved high accuracy of image recognition in an ANN simulation, which is promising for neuromorphic AI applications. Liang et al.^[^ ^170^ ^]^ reported an optoelectronic heterojunction FET of inkjet‐printed electrodes with a photoactive layer (Figure 4c). This device showed programmable conductance for short‐term and long‐term memory, as well as light‐controlled learning and forgetting, simulated as a backpropagation ANN. This study showed the advances of AM‐based optoelectronic neuromorphic devices as artificial e‐vision.

Inkjet printing of memristors has emerged in recent years for energy‐efficient neuromorphic hardware. Yoon et al.^[^ ^171^ ^]^ reported an inkjet‐printed Ag/Ag: Ag_2_O/Ag memristor via electrochemical filamentary conduction to achieve bipolar switching with a 10^8^ ON/OFF ratio. Franco et al.^[^ ^39^ ^]^ reported a fully inkjet‐printed Ag/IGZO/Ag memristor and showed layer thickness affected RS and memory windows (bipolar RS at lower thickness to threshold RS at higher thickness) (Figure 4d). Similarly, memristors based on TiO_2_,^[^ ^172^ ^]^ WO_x_,^[^ ^173^ ^]^ ZnO,^[^ ^174^, ^175^ ^]^ and h‐BN 2D materials^[^ ^42^, ^144^ ^]^ have been inkjet‐printed and all showed promising memristive properties despite varied compositions and RS modes.

**Figure 5:**
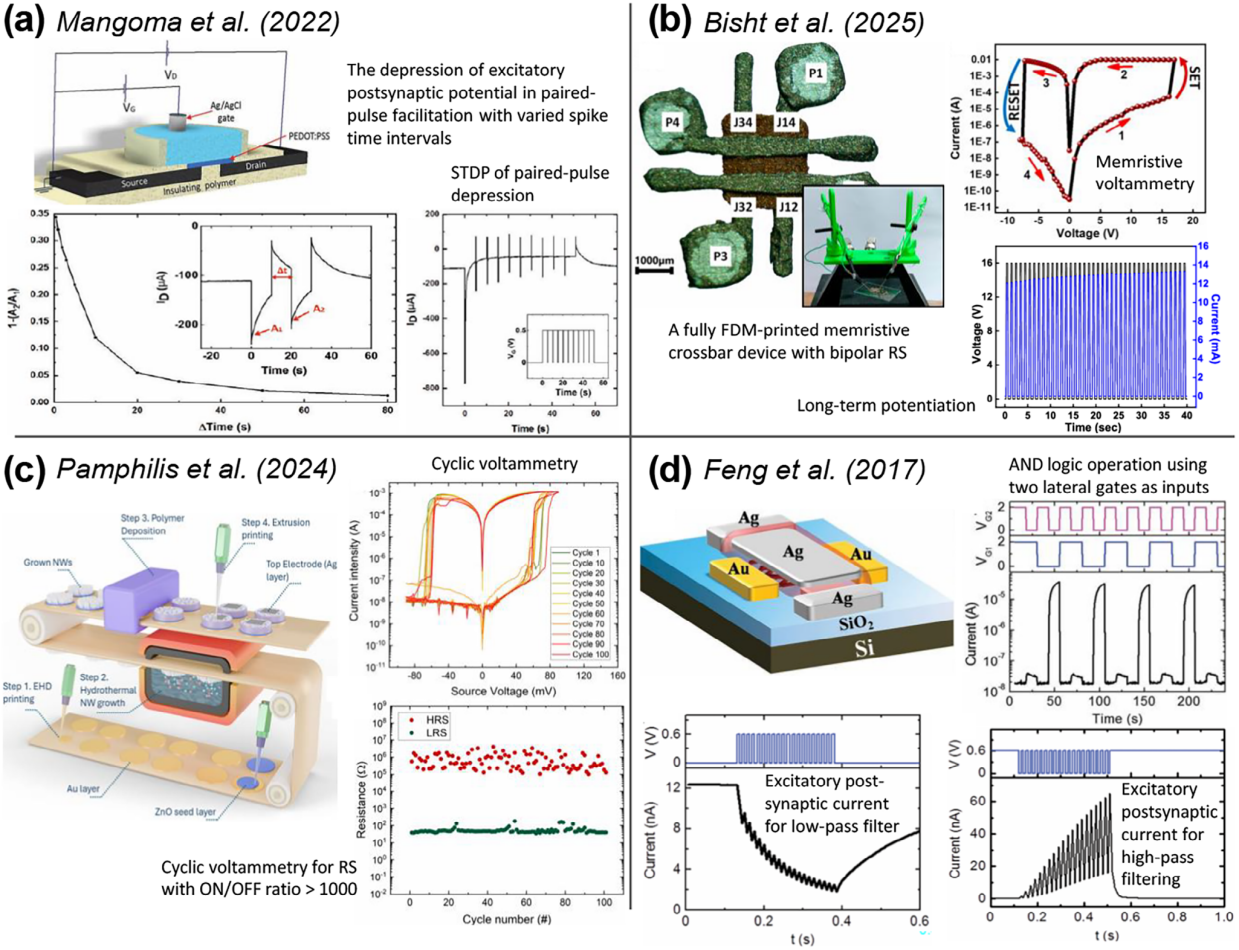
Examples of hybrid AM of transistors and memristors. a) A synaptic OECT produced via extrusion and inkjet printing, reproduced under the terms of the Creative Commons CC BY license.^[^ ^176^ ^]^ Copyright 2022 Wiley‐VCH. b) A fully FDM‐printed memristor, reproduced with permission.^[^ ^177^ ^]^ Copyright 2025 ACS. c) A memristor with electrodes processed using EHDA and direct ink writing, reproduced under the terms of the CC‐BY‐NC‐ND 4.0.^[^ ^179^ ^]^ Copyright 2024 ACS. d) A synaptic EDLT produced using aerosol jet printing and patterning, reproduced with permission.^[^ ^137^ ^]^ Copyright 2017 Wiley‐VCH.

Extrusion‐AM has served as a complementary method for synaptic electronics. Mangoma et al.^[^ ^176^ ^]^ reported a synaptic OECT with extrusion‐printed carbon‐filled polylactic acid as electrodes and polyester dielectrics, combined with an inkjet‐printed PEDOT: PSS semiconductor (**Figure** **5**a). They tested paired‐pulse depression and STDP, despite the large size of the device (channel length 3 mm, width 10 mm, extrusion width 0.35 mm, extrusion thickness 0.06 mm). Bisht et al.^[^ ^177^ ^]^ reported fully FDM‐printed memristors based on polylactic acid‐Cu composites (thickness ≈ 100 µm) with bipolar switching and synaptic learning (Figure 5b). Fan et al.^[^ ^178^ ^]^ reported an OECT via direct ink writing of Ag electrodes (line width ≈ 175 µm, thickness ≈ 20 µm) and a PEDOT: PSS channel, plus extruded polylactic acid insulators (thickness ≈ 7.5 µm). This OECT exhibited high transconductance and ion‐sensitivity. Collectively, these studies highlight the feasibility and low‐cost extrusion‐based printing.

PBF has been used as a post‐processing to supplement inkjet printing. Ko et al.^[^ ^140^ ^]^ generated all‐inkjet‐printed organic FETs by using PBF of Ag nanoparticle electrodes (width ≈ 6 µm). In another study,^[^ ^140^ ^]^ PBF and laser ablation were applied to create the semiconductor channels (length >1.2 µm and width >80 µm) of FETs.

Electrohydrodynamic atomization (EHDA) and aerosol jet printing are material jetting technologies with different jetting mechanisms (electric field and ultrasonic atomization respectively). They can achieve higher resolution and finer feature sizes, promising for microelectronics. Rehman et al.^[^ ^138^ ^]^ reported organic bistable non‐volatile memory devices via EHDA and screen printing, and tested bilayer structures to have higher RS efficacy than bulk‐heterojunction structures. Pamphilis et al.^[^ ^179^ ^]^ reported a ZnO‐nanowire memristor with EHDA of bottom electrodes and direct ink writing of top electrodes (Figure 5c). Their device showed non‐volatile bipolar RS at ultra‐low voltage. Xu et al.^[^ ^180^ ^]^ reported an EHDA‐printed Nafion memristor with threshold RS to mimic leaky integration‐and‐firing. Feng et al.^[^ ^137^ ^]^ reported a carbon‐nanotube‐based EDLT using aerosol jet printing which showed short‐term memory and filtering effects as artificial synapses (Figure 5d).

Stereolithography presents copious challenges for fabricating synaptic electronics; however, Bertana et al.^[^ ^181^ ^]^ recently reported VPP‐printed PEDOT: PSS with electroplated metal coating and transistor transfer behaviors. This work showed that metallization conductivity is not the major challenge in VPP instead, process control and technology integration are the key areas to be addressed.

#### 3.2.2 Analysis of AM‐Based Neuromorphic Electronics

**Figure 6:**
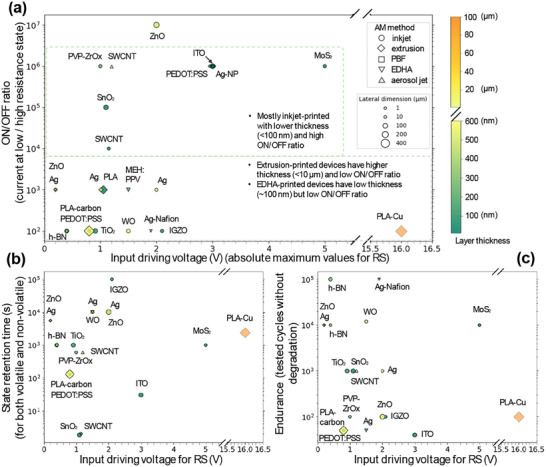
Analysis of AM‐based neuromorphic electronics with varied materials, processing techniques, printing resolutions, and synaptic performance. The input driving voltage (absolute maximum voltage for RS) versus a) ON/OFF ratios, b) state retention time, and c) endurance.

We summarized the AM‐based neuromorphic electronics from the literature with varied materials, AM methods, print resolution, and device performance (a detailed summary table in Supporting Data). Overall, for inkjet‐printed devices, lower layer thickness (<100 nm), narrower lateral dimensions, and higher ON/OFF ratios (<100 µm) were reported, whereas for extrusion and EDHA, ON/OFF ratios were generally lower (**Figure** **6**a). This can be attributed to thicker layers and wider extruded filaments in extrusion and EDHA, where charge carrier mobility reduces.^[^ ^39^ ^]^ Notably, inkjet‐printed inorganic and hybrid materials (MoS₂, TiO₂, ZnO, IGZO, Ag nanoparticles) exhibited relatively low driving voltages (typically ≈<3 V) and consistently high ON/OFF ratios (up to 10^7^), which are ideal for low‐power neuromorphic systems. Organic‐based devices (e.g., PEDOT: PSS, PLA composites) typically exhibited lower ON/OFF ratios (100–10 000) and variable driving voltages (0.5–16 V). This underlines the need to overcome resolution issues of extrusion‐AM for practical applications.

Inkjet‐printed inorganic‐material devices typically exhibited higher retention (up to 10 000 s) and endurance (up to 10^5^ cycles) (Figure 6b,c). These robust devices suit stable, long‐term synaptic applications. Organic and polymer‐based devices tend to have significantly lower retention (130–2400 s) and endurance (50–1000 cycles), which are suitable for volatile memories.

Note that there is insufficient literature for conclusive findings on variable correlations, nor for assessing their reproducibility, especially when AM methods, process, and testing conditions are all massively varied. Even the same materials and AM methods could have very different results. For example, inkjet‐printed ZnO memristors were reported to have unipolar RS (very high ON/OFF ratio)^[^ ^174^ ^]^ and threshold RS (low ON/OFF ratio).^[^ ^175^ ^]^ There are significant property differences for metal‐oxide memristors based on Ag‐nanoparticle electrodes.^[^ ^171^, ^172^, ^175^ ^]^ Hence, we encourage future research to explicitly state methodologies and develop parametric studies on material formulations – printing processes – property characterizations.

### 3.3 AM of Mechanical Neuromorphic Systems

**Figure 7:**
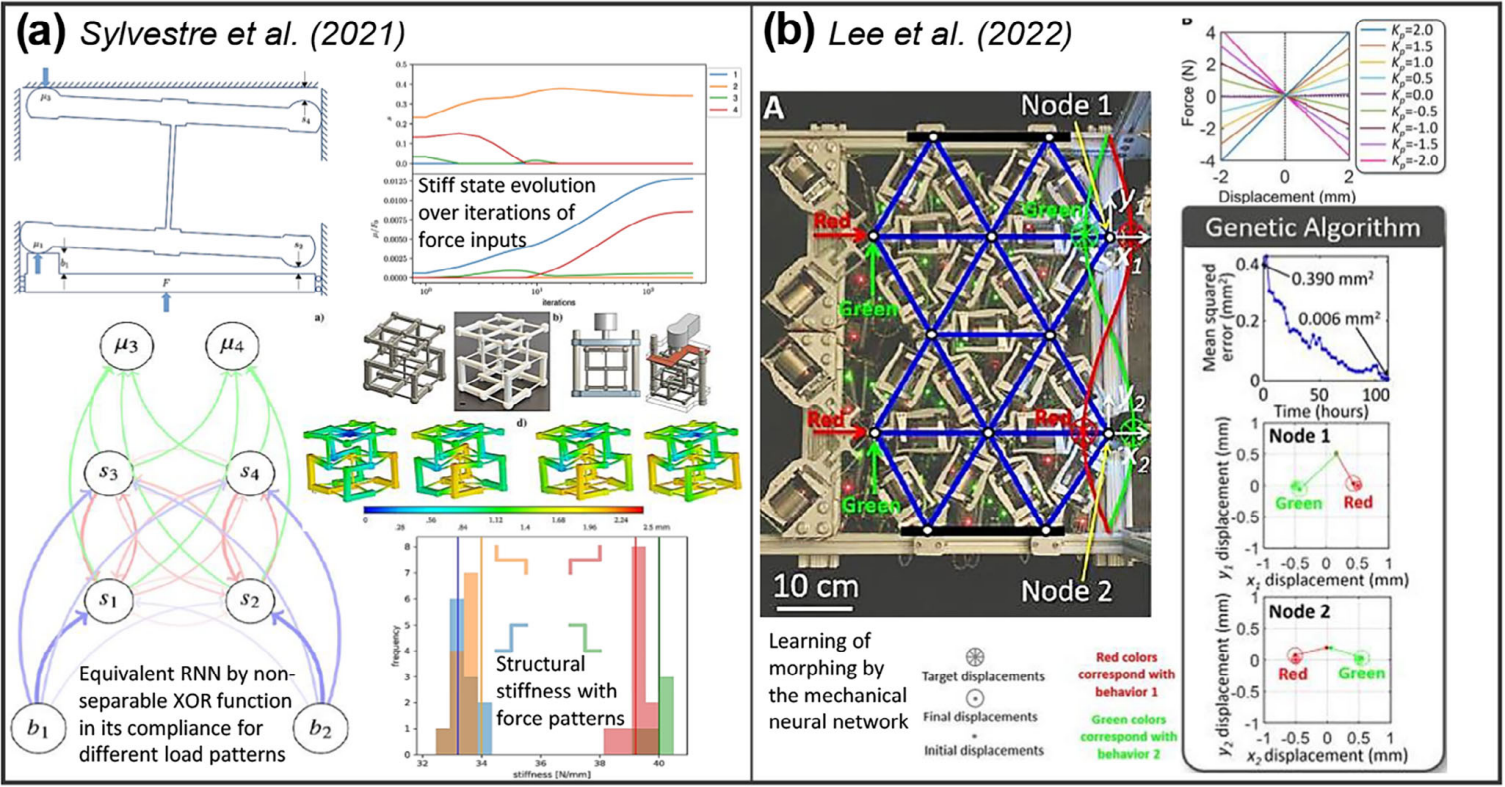
Examples of AM‐based mechanical neuromorphic systems. a) A 3D‐printed neuromorphic system based on one‐side‐contact nonlinearity used as recurrent neural networks, reproduced with permission.^[^ ^94^ ^]^ Copyright 2021 Elsevier. b) Mechanical neural networks with tunable beams of varied axial stiffness, reproduced with permission.^[^ ^182^ ^]^ Copyright 2022 AAAS.

Sylvestre et al.^[^ ^94^ ^]^ reported an AM‐based neuromorphic system based on one‐side‐contact nonlinearity (nodes connected by flexible prismatic beams) equivalent to recurrent neural networks. By varying the stiffness with gaps and reaction loads in response to applied loads, these cellular metamaterials optimized by using gradient descent training could perform tasks such as classifying patterns and XOR functions (**Figure** **7**a). Lee et al.^[^ ^182^ ^]^ reported a mechanical neural network consisting of tunable beams with closed‐loop control to achieve variable axial stiffnesses (linear and nonlinear) (**Figure** 7b). Lattice networks of the beams with active control enabled learning and exhibiting two distinct morphing behaviors. Learning and optimization are implemented through genetic algorithms and partial pattern search, validating these autonomous, adaptive, self‐learning mechanical systems.

These studies show the immense potential of AM‐based mechanical neuromorphic hardware which does not require prohibitive nanofabrication costs while achieving adaptive learning and computation. The embodiment of mechanical neural networks expands the field of neuromorphic systems and provides potential alternatives to electronics in harsh environments or where electricity is deficient. Current research on physical computing has only just begun. Since they hold a great promise for scalable, energy‐saving low‐cost mass‐production and environmental sustainability, we believe that it is worthwhile exploring other structures (e.g., 3D lattices, auxetic, multistable) and materials (e.g., piezoelectric, optoelectronic, biomaterials) for neuromorphic mechanical intelligence.

## 4 Opportunities and Challenges

### 4.1 Applications in Intelligent Systems

Functional neuromorphic hardware is at the frontier of AI and advanced computational systems with robust applications such as neuromorphic sensing, bionics, and robotics.^[^ ^70^ ^]^ Neuromorphic sensing integrates sensors and neuromorphic systems for event‐based analog computation (only process data when there is a change in the sensory input). This is fundamentally different from ANN computation by graphics processing units, which persistently process data with high latency and convert analog input into digital formats before processing.^[^ ^183^ ^]^

Neuromorphic sensing benefits from stimuli‐responsive actuation and memory effects of synaptic devices. Synaptic transistors can support artificial perception by processing environment inputs (e.g., images, light intensity) and can be further integrated in ANNs to achieve real‐time sensing.^[^ ^184^ ^]^ Neuromorphic cameras based on pixel‐light intensity and frequency‐detection algorithms can be used to detect drones and events.^[^ ^185^ ^]^ This is particularly useful for autonomous driving (self‐driving vehicles). Chen et al.^[^ ^184^ ^]^ reviewed event‐based neuromorphic vision sensors that capture pixel changes to produce asynchronous event streams. Algorithms such as spatial‐temporal correlation filters and spatial‐temporal encoding can be applied for real‐time visual perception. Hwu et al.^[^ ^24^ ^]^ demonstrated a novel self‐driving robot based on deep convolutional neural networks implemented on IBM TrueNorth neuromorphic chips. Based on the motor command dataset from manually controlled robots and video recording, the self‐driving robot traversed steep paths in real time under closed‐loop control. Paredes‐Vallés et al.^[^ ^186^ ^]^ presented a fully neuromorphic vision‐to‐control pipeline for autonomous drone flight by implementing SNN on event‐based cameras. These studies mark a significant milestone in neuromorphic autonomous navigation.

**Figure 8:**
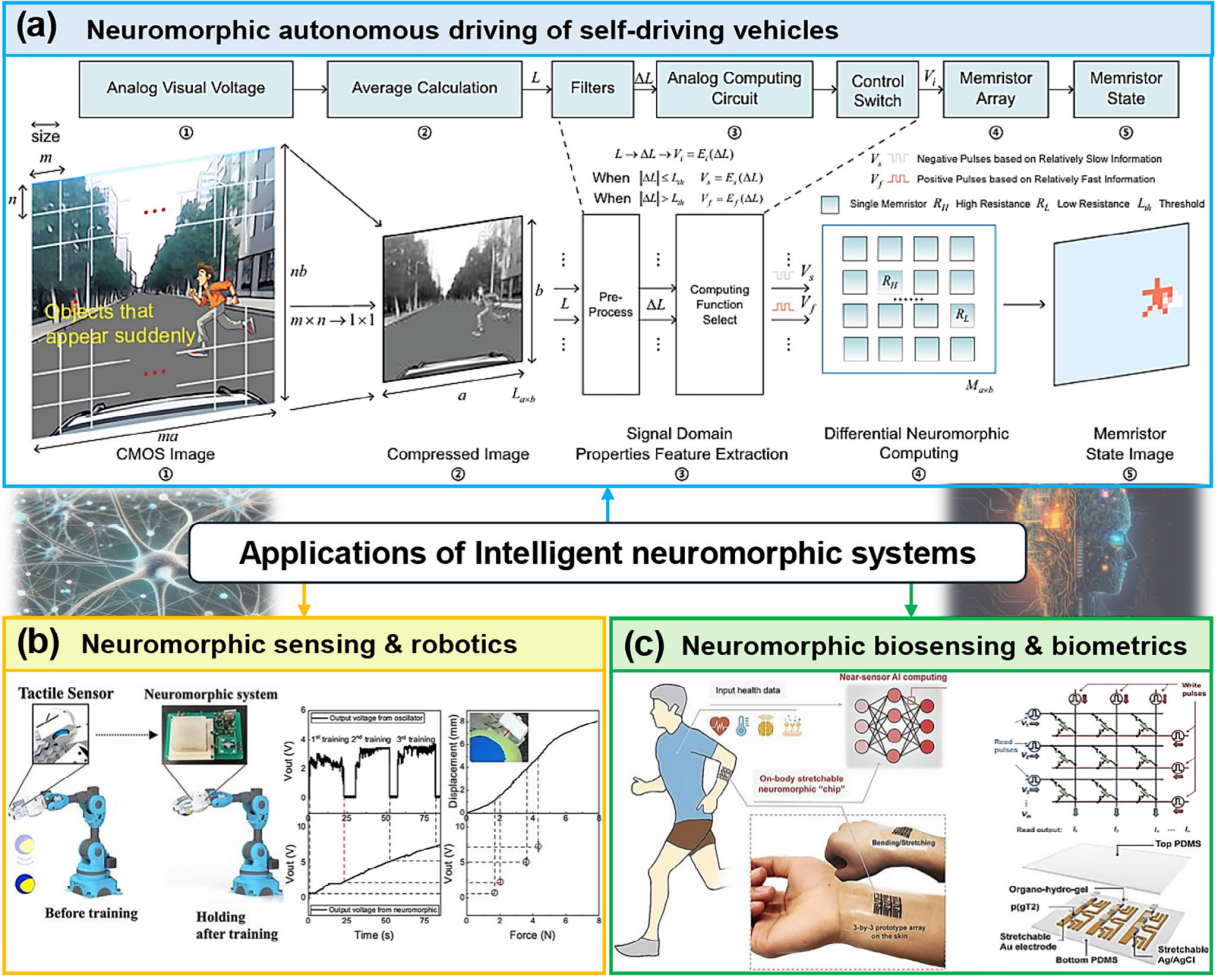
The applications of neuromorphic systems in a) autonomous driving by synaptic memristor crossbar arrays, reproduced under the terms of the Creative Commons CC BY license.^[^ ^187^ ^]^ Copyright 2024 Springer Nature. b) Sensing and robotics by a neuromorphic chip‐controlled humanoid hand, reproduced with permission.^[^ ^188^ ^]^ Copyright 2022 Elsevier. c) Biosensing and biometrics by a synaptic transistor for electrocardiogram monitoring, reproduced with permission.^[^ ^190^ ^]^ Copyright 2022 Elsevier.

On the other hand, crossbar arrays of artificial synapses also enable neuromorphic sensing. Wang et al.^[^ ^187^ ^]^ proposed neuromorphic computing based on memristor arrays for differential signal processing and online adaptation to sensory stimuli. They demonstrated two main applications: robot hand gripping with adaptive force based on tactile feedback and autonomous driving, where a memristor array extracts decision‐making data from unstructured environments with high accuracy (**Figure** 8a). A similar system has been reported by Bao et al.,^[^ ^188^ ^]^ where a neuromorphic humanoid hand performed gripping with spontaneously adjustable forces by neuromorphic signal transmission and processing without spike‐based algorithms (Figure 8b). This was achieved by integrating AM‐based origami‐structured pressure sensors and a portable OECT with non‐volatile memory to mimic reflex arcs. Similarly, Eom et al.^[^ ^189^ ^]^ integrated neuromorphic circuits based on memristors and FETs into AM‐based soft robots to achieve tactile sensing and distributed computing. This study presents the integration of neuromorphic hardware in 3D‐printed robots, promising for AI robotics.

Biosensing and biometrics can benefit from AM‐based neuromorphic hardware. Organic synaptic transistors and memristors based on biodegradable and bioactive materials can be programmed to real‐time monitor bio‐behaviors and integrated in wearable devices. Dai et al.^[^ ^190^ ^]^ developed durable neuromorphic OECTs with excellent synaptic behaviors for electrocardiogram monitoring (**Figure** 8c). Other studies showed neuromorphic biosensing of glucose,^[^ ^191^ ^]^ toxic gas,^[^ ^38^ ^]^ and viruses.^[^ ^192^ ^]^

AM‐based neuromorphic devices have not yet been developed in system‐scale applications, and current studies mostly focus on optimization of materials, processing, and properties of single‐node units (artificial synapses) in lab‐scale, while only a few demonstrated 3 × 3 memristor arrays,^[^ ^175^ ^]^ inkjet‐printed FET in integrated circuits,^[^ ^43^ ^]^ and transistor arrays (>900 units).^[^ ^164^ ^]^ Since the crossover of AM and neuromorphic engineering just began, we expect to see more scale‐up systems, as AM technology innovates and mellows. Several critical aspects of AM‐based neuromorphic hardware must be considered, which are discussed next.

### 4.2 Challenges for AM‐Based Neuromorphic Hardware

- Materials: Material functionality is the key to allowing advanced neuromorphic computing. For synaptic electronics, electrodes should be highly conductive to lower the electrical impedance and power consumption. Metal nanoparticles, especially Ag nanoparticles, have been introduced to act as electrodes to sufficiently lower the driving voltage. Organic electrodes composed of conductive polymers (e.g., PEDOT: PSS, polyaniline, poly(3‐hexylthiophene)) are more compatible and feasible with extrusion‐based AM, though they require more elaborate chemical formulation. Additionally, carbon‐based materials (e.g., carbon nanotubes, graphene oxide, carbon nanomaterials)^[^ ^193^ ^]^ could be processed into electrode layers using extrusion‐based and laser‐based AM. There are more choices available for dielectric materials, ranging from metal oxides to ionic polymers, some of which are 3D‐printable. Hydrogel dielectric materials with high ionic mobility could be used in sustainable synaptic electronics for bionics and biosensing^[^ ^194^, ^195^, ^196^, ^197^ ^]^ (e.g., glucose oxidase dielectric materials to detect glucose,^[^ ^198^, ^199^, ^200^, ^201^ ^]^ ionogels for piezo‐electronics,^[^ ^202^ ^]^ organic ferroelectrics,^[^ ^203^ ^]^ self‐healing hydrogels).^[^ ^204^ ^]^ Photoactive and chemosensitive dielectrics can be used for functional visual and sensing systems (e.g., molecules of enol–keto photo‐tautomerization,^[^ ^205^ ^]^ NiO,^[^ ^206^ ^]^ and upconverting nanoparticles,^[^ ^207^ ^]^ NO_2_‐sensitive ionogels to mimic the olfactory system).^[^ ^38^ ^]^ Materials with complementary properties can facilitate AM processes. For example, similar melting points or rheology for dielectric materials and conductive materials may facilitate multimaterial construction in material extrusion and jetting, either for the use of a single method or a hybrid process.
- Precision and resolution: Submicron precision is an exigent prequisite for AM to process synaptic devices. Extrusion‐based AM is mostly limited by the nozzle size and material rheology. In some cases, mechanical and thermal actuation may be insufficient to deposit materials in submicron volumes due to electrostaticity and viscoelasticity. Modifications including EHDA actuation or sputtering, which are compatible with extrusion AM, could be considered to assist material deposition in high resolution, accompanied with miniaturized nozzles. Nanomaterials such as nanoparticles, 2D materials, MXene^[^ ^79^ ^]^ could be used to achieve higher layer resolution. Inkjet printing with fine‐sized nozzles facilitates the precise deposition of nanoparticle solution and rapid solidification. Similarly, nanoparticles can be encapsulated in resin matrices and 3D printed via extrusion, VPP, or PBF. Postprocessing (e.g., pyrolysis, gasification, sintering) may also be employed; however, the distribution, size, and content of the nanoparticles should be optimized to avoid affecting the printing performance. One advantage of extrusion and jetting is the orientation of particles or macromolecules caused by shear and directional printing. This feature could be exploited for orientation‐dependent RS (e.g., polar‐electret molecule orientation facilitated by microfluidic inkjet printing)^[^ ^169^ ^]^ and 2D material orientation for more controlled RS.
- Stability: For these AM‐based electronics, long‐term stability over extended cycles of electrical stimuli, environmental stressors, and thermal fluctuations is not well understood. Repeated electrical cycling may amplify RS hysteresis effects, which can shift or narrow the threshold for RS and thus reduce the memory window. Cyclic stress may also introduce additional lattice defects (e.g., dislocations, vacancies, or interstitials) which could impair electron transport and RS efficiency. Cracking and delamination may occur as a result of electromigration and thermal effects over cyclic voltage. These issues could lead to structural failure, imprecise synaptic behaviors, and computation errors. Flexible bioelectronics have been studied,^[^ ^190^, ^208^, ^209^ ^]^ highlighting the advantages of organic materials (strain‐independent conductivity and self‐healing polymers)^[^ ^210^ ^]^ and how strain could be utilized in neuromorphic sign‐language sensing.^[^ ^45^ ^]^ The combination of inorganic neuromorphic devices and soft organic modules also allows good mechanical flexibility. These stretchable electronics provide stable tissue‐device interfacing for wearable bioelectronics. Long‐term stability requires rational modeling of materials, architecture design, and environmental protection.
- Scalability: the layer‐by‐layer nature of AM results in a considerably lower throughput compared to photolithography. Theoretically, creating a hybrid system with multiple deposition tools and conventional techniques for repetitive patterning of structures may increase the production rate. However, achieving the high‐density integration for complex neuromorphic systems is difficult due to the resolution limits of current AM technologies. This is also constrained by the availability and compatibility of functional materials that meet both performance and manufacturing requirements. The economic feasibility of scaling up is another critical concern, as the cost of advanced modified AM equipment and low throughput may outweigh the benefits of customization and flexibility. Addressing the scalability issue depends on the development of high‐throughput AM technologies with low cost, improved material formulations, enhanced precision, and better integration with existing manufacturing workflows.

## 5 Conclusion 

We reviewed additive manufacturing technologies in the context of neuromorphic systems regarding technical advances, materials, and applications. Additive manufacturing allows the rapid production of neuromorphic hardware with high resolution, scalability, and customizability but low cost. Among various techniques, inkjet printing is currently the most suitable choice for fabricating neuromorphic hardware, due to its high resolution, material flexibility, and processing compatibility. Inkjet‐printed synaptic transistors based on photoactive materials have been explored as artificial vision systems, proving high‐accuracy neuromorphic computing akin to ANNs. Material extrusion and selective laser sintering could be useful as supplementary methods, while resolution and precision limit their wide applicability. Vat photopolymerization has good resolutions, but both the process and materials will require substantial modification to be compatible for neuromorphic devices. In addition, mechanical neuromorphic systems have emerged in recent years as an appealing replacement for energy‐consuming electronics to realize computation based on mechanical loading and interactions. Undoubtedly, additive manufacturing may propel the development of mechanical neuromorphic hardware and create new computation paradigms in mechanical intelligence. It may also facilitate the construction of 3D neuromorphic architectures for next‐generation computing systems.

While additive manufacturing offers unparalleled design flexibility and material versatility, the transition from laboratory‐scale fabrication to large‐scale commercialization introduces hurdles across multiple dimensions. Challenges including RS efficacy, stability, scalability, and system compatibility are to be addressed, while AM resolution and materials will undoubtedly improve with continuous innovations. AM‐based neuromorphic hardware can be used as high‐efficiency low‐cost computation systems, promising for real‐time sensing, cognitive AI, and data analysis. Collaborative efforts among material science, engineering, neurobiology, and computer science will be critical to unlocking the full potential of AM in neuromorphic engineering.

## 6 Supplementary Data

**Table** **2** shows the data summary of AM‐based synaptic transistors and memristors in terms of their printing resolutions and synaptic performance.

**Table 2:** Literature summary of AM‐based synaptic transistors and memristors.

AM method	Printed materials	Layer thickness	Lateral dimension [µm]	Driving Voltage [V]	ON/OFF ratio	Retention [s]	Endurance (tested cycles)	Ref.
inkjet	Ag NP	30 nm	50	1.5–3	10^6^	/	/	[154]
SLS			1					
inkjet	Ag NP	30 nm	2	−3–3	10^6^	/	/	[157]
inkjet	Ag NP	500 nm	25	−1–2	10^3^	10^4^	10^3^	[171]
inkjet	MoS_2_	20 nm	30	0–5	10^6^	10^3^	10^4^	[43]
inkjet	SnO_2_, ITO	12 nm	100	−1–1	10^5^	1.84	10^3^	[170]
inkjet	ITO	15 nm	100	0—3	10^6^	30	40	[37]
inkjet	PVP‐ZrOx	200 nm	30	−1–1	10^6^	600	100	[169]
inkjet	IGZO	141 µm	30	−1.5–2	100	10^5^	100	[39]
inkjet	TiO_2_	80 nm	90	−1–1	100	10^3^	10^3^	[172]
inkjet	h‐BN	250 nm	60	−0.4–0.4	100	10^3^	10^5^	[42]
inkjet	h‐BN	250 nm	20	−0.3–0.4	100	10^3^	10^4^	[144]
inkjet	WO	600 nm	50	−0.5–1.5	100	10^4^	1.2 × 10^4^	[173]
inkjet	ZnO	500 nm	150	0–2	10^7^	10^4^	100	[174]
inkjet	ZnO NP	/	200	−1–1	/	0.1	100	[175]
inkjet	PEDOT:PSS	30 nm	2	1.5–3	10^5^	/	/	[162]
inkjet	SWCNT	10 nm	20	−1–1	10^4^	2	/	[49]
inkjet	PEDOT: PSS	10 nm	15	0.5–0.8	100	130	50	[176]
FDM	PLA‐carbon	60 µm	300					
FDM	PLA‐Cu	100 µm	400	−8–16	100	2.4 × 10^3^	100	[177]
DIW	Ag nanoparticles	20 µm	175	0–1	10^3^	/	/	[178]
FDM	PLA	7.1 µm	20					
EDHA	ZnO	1.3 µm	1	−0.2–0.2	10^3^	5.5 × 10^3^	10^4^	[179]
extrusion	Ag electrode	/	5					
EDHA	MEH: PPV	200 nm	50	−1.5–1.5	10^3^	10 000	50	[138]
EDHA	Ag‐Nafion	200 nm	6	0–2	100	/	10^5^	[180]
aerosol jet	SWCNT	1 µm	20	−1.2–1	10^6^	600	10^3^	[137]

## Conflict of Interest

The authors declare no conflict of interest.
